# Overview of nomenclature and diagnosis of amyotrophic lateral sclerosis

**DOI:** 10.1080/07853890.2024.2422572

**Published:** 2024-10-29

**Authors:** Renshi Xu

**Affiliations:** Department of Neurology, Jiangxi Provincial People’s Hospital, Clinical College of Nanchang Medical College, First Affiliated Hospital of Nanchang Medical College, National Regional Medical Center for Neurological Diseases, Xiangya Hospital of Central South University Jiangxi Hospital, Nanchang, Jiangxi, China

**Keywords:** Amyotrophic lateral sclerosis, motor neuron disease, nomenclature, diagnosis, insight, doubt

## Abstract

The nomenclature of amyotrophic lateral sclerosis (ALS) currently is blurred, indistinct and no accurate and haven’t been properly updated since the first description, which is far from being suitable for the current implementation of clinical practise and scientific research of ALS, and urgently need an solution. Furthermore, the current diagnostic criteria need also further been improved, because the current clinical diagnosis of ALS majorly depends on the clinical manifestations yet. Up to now, no any objective clinical auxiliary examination can be helpful to diagnose ALS besides the electromyogram identifying the lower motor neuron damage, which isn’t conducive to early diagnosis and prolongs the time of ALS confirmed diagnosis. In this mini review, we discussed the current doubt about the nomenclature and diagnostic criteria of ALS, and prospected in order to further improve and normalize the nomenclature and diagnosis of ALS.

## 1. Introduction

Amyotrophic lateral sclerosis (ALS) is a rare neurodegenerative disease, usually classified into the familial and sporadic ALS clinically based on the genetic background. The current nomenclature of ALS is considered to be not accurate and proper based on the current results of clinical and experimental studies [1,2]. At present, there exists the different nomenclature in the different countries or/and regions according to their bias. For example, ALS and motor neuron diseases (MNDs) are usually considered as the same nomenclature or disease name on the course of clinical diagnosis and treatment in the countries of the United Kingdom. However, in the other countries and regions of no United Kingdom such as the United States, the partial European countries and China, MNDs are usually are categorized into four different subtypes including ALS, primary lateral sclerosis (PLS), progressive muscular atrophy (PMA) and progressive bulbar palsy (PBP), ALS is considered to be one common subtype of MNDs [3–6]. In fact, more and more evidences demonstrate that MNDs are belong to a wide spectrum of disorders, ALS is the most common MNDs, while PLS, PMA, PBP are the most common clinical phenotypes of this heterogeneous disease. Other MNDs include spinobulbar muscular atrophy, infectious MNDs, spinal muscular atrophy, etc. The current nomenclature about ALS in the worldwide hasn’t reached a consensus yet. Therefore, the ambiguous nomenclature about ALS seriously affects and hinders the conduction of clinical diagnostic and treatment as well as various clinical investigations and trials.

In addition, the current diagnostic criteria of ALS [6,7] also exist some deficiencies. Because the objective clinical auxiliary examination measures for diagnosing ALS currently are relatively insufficient, the newest revised diagnostic criteria [6,7] still conduct the clinical diagnosis mainly depended on the clinical manifestations. Among the current diagnostic criteria [6,7], besides the electromyogram can be used in defining the motor neurons damage in anterior horn of spinal cord, other auxiliary examinations such the routine magnetic resonance imaging of brain and spinal cord are only applied in the differential diagnosis of some ALS-mimicking diseases [8]. Therefore, it is challenging to diagnose ALS, and is very difficult to catch the disease and conduct the confirmed diagnosis early on the course of ALS depending on the current diagnostic criteria [6,7], which delays the confirmed diagnostic time, prevents the early intervene of ALS, and isn’t benefit to conduct the clinical investigation on the pathogenesis, etiology and treatment of ALS at the early stage yet [9,10]. The main reasons bring the difficulty of early confirmed diagnosis are the heterogeneity of clinical phenotype of ALS, which need exclude lots of curable ALS-mimicking diseases at the early stage of ALS, thus need spend a lot of time to perform the extensive related clinical auxiliary examination including the laboratory test, genetics screening, neuroimaging, etc. because of lacking the confident diagnostic biomarkers at present [11].

In view of the current situation about the nomenclature and diagnostic criteria of ALS [8,12], it is necessary to update them as soon as possible after being comprehensively discussed by the neurologists focusing on studying ALS. Especially in the nomenclature of ALS, they aren’t very suited to the clinical and investigating application of neurologists. It is urgently necessary to make an optimal nomenclature for this disease because the no accurate nomenclature will seriously hinder the progression of the clinical and investigating practice in this disease. To this end, this topic should be more thoroughly discussed.

**Figure 1.**
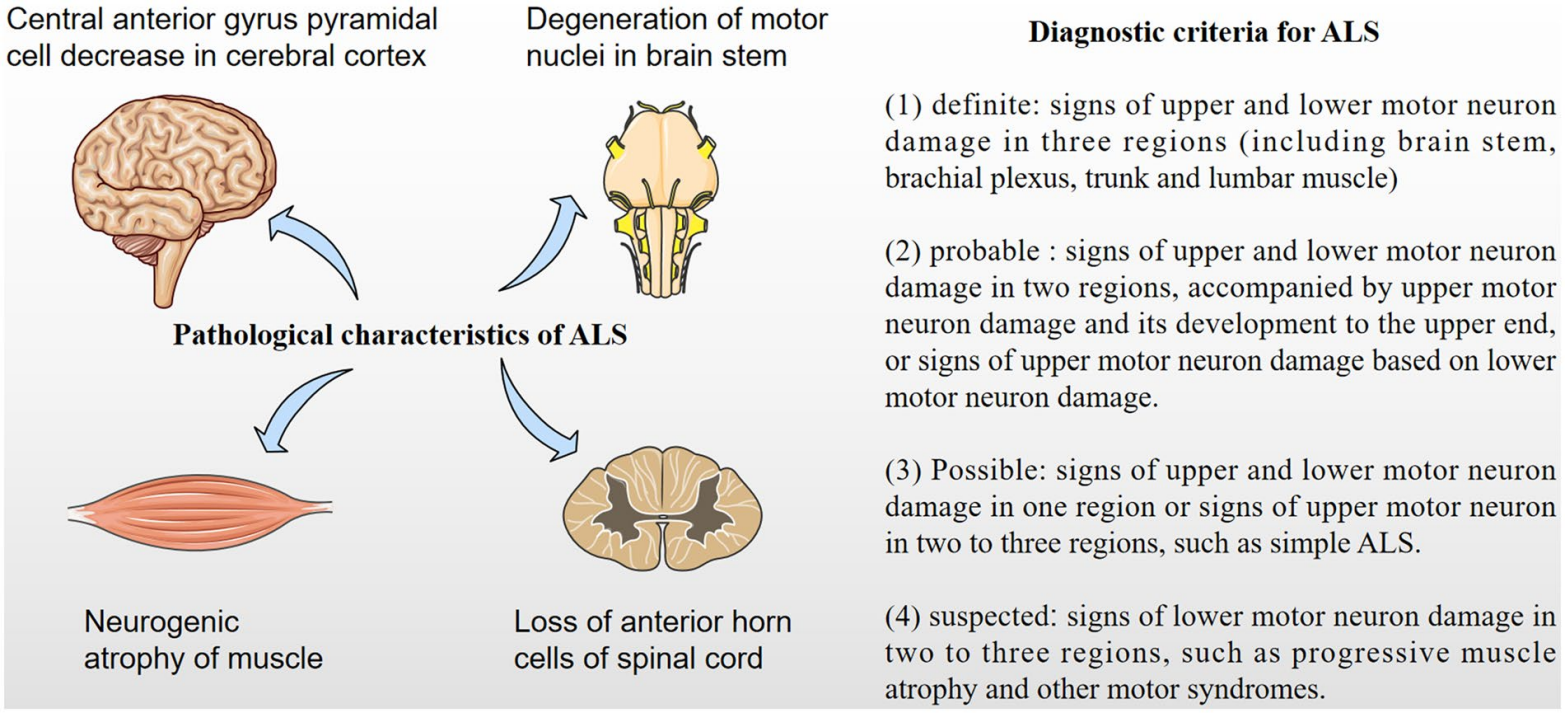
Schematic representation of ALS classic pathological characteristics and diagnostic criteria. (A) The classic pathological characteristics of ALS were thought to only involve in the motor neurons in cerebrum, brain stem and spinal cord, including pyramidal cells in the central anterior gyrus of cerebrum, motor nuclei in brain stem, anterior horn cells in spinal cord. (B) The classic diagnostic criteria for ALS is necessary to consist of the symptoms and signs of upper and lower motor neuron damage including the clinical diagnostic definite, probable, possible and suspected ALS patients.

The goal of this mini review paper is to discuss the possible existed question about the current nomenclature and diagnosis of ALS [2]. In our clinical and investigated practise, we found that the current nomenclature and diagnosis of ALS (Figure 1) exited some questions and doubts. This mini review aims to emphasis and further promote our idea in our previous published paper about ‘considerations on the concept, definition, and diagnosis of amyotrophic lateral sclerosis’ again [2], make our thoughts receive more extensive attention from more neuroscientists and resolve the current issues about the nomenclature and diagnosis of ALS as soon as possible.

## 2. Search strategy

We searched the current ALS nomenclature and diagnosis information on PubMed, EmBase, Google Scholar, WanFang, Cochrane Library websites and various medical guidelines using the key search terminology ‘amyotrophic lateral sclerosis’, ‘motor neuron disease’, ‘familial amyotrophic lateral sclerosis’, ‘sporadic amyotrophic lateral sclerosis’, ‘ALS’, ‘familial ALS’, ‘sporadic ALS’, in combination with ‘nomenclature’, and ‘diagnosis’ at the end of February 2024. In addition, we used the modifications of main keywords to thoroughly search the literature on the history, current nomenclature and diagnosis of ALS. We also analyzed the current problems related to these information based on the retrieved literature as well as the difficulties that we encountered in our clinical work. The literature review was performed with the strict control of information based on the assembled problems and the information from searching literature. The problems and proposals extracted from our literature review and clinical experiences are presented herein [2]. These related articles were included the articles from the reference lists, the review articles, the major textbook chapters as well as the abstracts and reports from the relevant meetings. The final reference lists were generated on the basis of originality and relevance to the topics covered in this commentary. Emphasis was placed on publications from the past nearly 30 years, but didn’t exclude the commonly referenced and highly regarded older publications.

## 3. The current nomenclature of ALS

ALS is a progression adult onset neurodegeneration disease, which mainly damages upper and lower motor neurons, partially also involves frontotemporal and other regions of brain. The extent to the different neuron cells is affected varies between individuals. The current phenotype classification of ALS are conducted based on the onset pattern, which results in confusion between nomenclature of ALS in the clinical work. The current nomenclature such as the international classification of diseases are the systematic approaches, while these approaches usually don’t consider some special characteristics in the clinical practice, such as progression, the genetic factors, the vast clinical variability of presentation and prognosis or the accurate pathological alterations. At present, the routine classification approach of disease might not be suitable for the nomenclature of ALS for neurologists, which will bring the difficult for their clinical and research work, such as the confused nomenclature, which leads the inaccurate diagnosis of ALS [9]. Moreover, the classification and nomenclature of this disease, whether sporadic or familial, also is confused [1,^12^]. A novel nomenclature being suitable for the complex and variable phenotype of ALS urgently need be setup in clinical practice.

### 3.1. Doubt about the nomenclature of ALS

In fact, ALS is a pathological nomenclature, especially about the nomenclature of lateral sclerosis, the lateral sclerosis is a definition of pathology, currently can’t find the lateral sclerosis signs by the clinical examination, only find the potential lateral damage. The typical pathological features of ALS are the irreversible chronic progressive and selective death of the upper and lower motor neurons which control the voluntary muscles in the cerebral, spinal and/or brain stem regions, and the damage change (Pathological sclerosis) of the corticospinal lateral tract and the cortical brainstem tract. Therefore, it is known as ALS [6,13,14]. In the meanwhile, the MNDs are a group of diseases of irreversible chronic progressive and selective death of UMD and/or LMD, traditionally artificially divided into four subtypes according to the clinical manifestations and the onset pattern, among them, ALS is thought to be the commonest subtype, the other three subtypes are PLS, PMA and PBP [3–6]. At present, the nomenclature of ALS and MNDs usually are confusedly used in the clinical and research work of ALS, which might bring some inaccurate factors and results for the diagnosis and treatment and trials or study of ALS. Firstly, the doubt about the ALS nomenclature is whether or not the other three subtypes of MNDs are the different onset types or the different disease stages of ALS, all MNDs might ultimately damage both UMD and LMD if we performed the pathological examination of brain and spinal cord for every ALS and MNDs patients diagnosed based on the clinical information. In other words, whether or not it may be considered that ALS and MNDs is an equal concept or disease or the PLS, PMA and PBP subtypes of MNDs is the different stages or onset pattern of ALS [15]. So far, there is no clear evidence whether or not the different subtypes of MNDs is an independent disease and has an independent pathogenesis, moreover, a key difficult question whether or not PLS, PMA and PBP is one distinct disease or one subtype of ALS spectrum also remains unclear [16–21]. Secondly, the doubt about the ALS nomenclature is that the current studies have determined that ALS doesn’t only damage the MNs, but also involves the more extensive damage of other neural systems besides MNs, even including the neuromuscular junction [22] and the cerebral marginal systems [23], leading some non-motor symptoms such as the cognition [24] and behaviors disorders [25].

ALS can be defined or take the concept as a group of neurodegenerative diseases featured by a progressive muscular paralysis caused by the degeneration of motor neurons in the primary motor cortex of cerebrum, the motor nuclei of brainstem and the motor neurons of the anterior horn in spinal cord. The nomenclature of amyotrophy refers to the atrophy of muscle fibers caused by the denervation contributing by the degenerate of anterior horn cells, which results in the weakness, atrophy and visible fasciculation of the implicated muscles. The nomenclature of lateral sclerosis refers to the pathologically harden of the corticospinal lateral tracts due to degenerate and replace by glial cells in these areas [11,26].

At present, based on our acknowledgments, the current disease name related to ALS covers ‘amyotrophic lateral sclerosis (ALS)’, ‘motor neurone disease (MND)’, ‘Charcot’s disease’ and ‘Lou Gehrig’s disease’. ALS is a nomenclature used to cover the spectrum of neurodegenerative syndromes characterized by the progressive degeneration of motor neurons. However, it is also the nomenclature used in the modern clinical practice to indicate the commonest form of this disease, the classical ALS (Charcot’s ALS). Other syndromes related to this spectrum of disorders include PLS, PMA, PBP, flail arm syndrome (Vulpian-Bernhardt syndrome), flail leg syndrome (Pseudopolyneuritic form) and the ALS with the multiple extramotor system involvement. The extramotor deficits in ALS including the cognitive and behavioral disorders. The cognitive and behavioral and language impairment in ALS has been known for more than a century [11,27]. The extra-pyramidal involvement is reported to involve in the ALS with frontotemporal dementia-spectrum disorders as early as 1963 [28]. At present, ALS has been now universally recognized as a complex multisystem disorder with a multiple and considerable extra-motor involvement [29]. Recent advances in neuroimaging have been determined the neuropsychological manifestations of frontotemporal, parietal and basal ganglia involvement in ALS, which has the important implications for the extra-motor involvement in ALS [30]. Lord Russell Brain proposed the nomenclature MNDs to incorporate these conditions into a single spectrum of disorders. The nomenclature ‘bulbar onset ALS’ and ‘spinal onset ALS’ have largely replaced the nomenclature PBP and Charcot’s ALS in current practice [11]. These syndromes share a common molecular and cellular pathology comprising of motor neurone degeneration and the presence of characteristic ubiquitin-immunoreactive intraneuronal inclusions of some special proteins including superoxide dismutase 1 [31], TAR DNA-binding protein 43 [32], fused in sarcoma/translated in liposarcoma [33] and C9orf72 [34].

Therefore, the MNDs nomenclature also is the same with ALS to be not far proper for the present clinical and research application, and to name ALS as MNDs is even more inappropriate yet. Based on the current situation of ALS nomenclature, the nomenclature of ALS and MND is far from suitable for naming this disease, and the nomenclature of both ALS and MNDs remain to further be ascertained, and urgently need been updated. According to the author’s clinical and research experiences, it is though that ‘Amyotrophic lateral sclerosis syndrome’ or ‘Amyotrophic lateral sclerosis spectrum diseases’ or ‘Motor neuron disease syndrome’ or ‘Motor neuron spectrum diseases’ might be the more suitable nomenclature whether in the clinical practice or in the scientific study [2].

## 4. The current diagnosis of ALS

The characterized clinical symptoms and signs of ALS patients include the combined lesion of chronic progressive upper motor neuron (UMN) and lower motor neuron (LMN), and these clinical manifestations can’t be explained by any other diseases. Meanwhile, it is necessary to combine the related evidences on neuroelectrophysiological, imaging, genetics, cerebrospinal fluid or serological laboratory examination and the patient’s history, achieves an adequate clinical diagnosis on ALS. Although the diagnostic criteria were multiple times revised following by the deeply ALS researching, the diagnosis of ALS still mainly depends on the characteristic clinical manifestations and the evidences excluding ‘ALS-mimic’ syndromes [8–10,11] (Table 1). For that, the current diagnostic criteria result in 5–10% diagnostic error ratio [35,36] and 10–16 months of diagnostic delay [37–39].

**Table 1.** The different diagnostic criteria, the pathological features, the sensitivity and specificity.

Nomenclature	Year	Location	Organization	Diagnostic criteria	Pathological features	Advantages and disadvantages
El Escorial criteria [1]	1994	El Escorial, Spain	World Federation of Neurology (WFN)	Suspected ALS: LMN signs only. Probable ALS: UMN and LMN signs in at least two regions, with some UMN signs rostral to LMN signs. Possible ALS: UMN and LMN signs in only one region, or UMN signs alone in two or more regions, or LMN signs rostral to UMN signs. Definite ALS: UMN and LMN signs in three regions of the body [1].	The combined degenerative damage of UMN and LMN dysfunction involving the bulbar, cervical, thoracic and lumbosacral segments. Sphincter and extraocular muscles are classically spared. Progresses in an anatomical manner from rostral to caudal. depending on the number of clinical affected segments, supplemented by neuroelectrophysiologicall (electromyography and central motor conduction time) and imaging (MRI, CT and PET) data to exclude ALS mimics. Classical ALS: Phenotype presents both LMN and UMN signs with muscle atrophy in limbs. Four levels of diagnostic certainty (probable, possible, suspected and definite ALS) [5–7].	Commonly used clinical diagnosis. Developed for research purposes, the absence of a confirmatory diagnostic test for ALS. The diagnostic sensitivity is very low, particularly in the early stages of the disease, resulting in substantial diagnostic delay and limiting recruitment of patients with ALS into therapeutic trials. The specificity of the criteria was the highest [5–7].
The revised El Escorial criteria or Airlie House criteria [2]	2000	Airlie House, Warrenton, VA, United States	WFN Research Committee on Motor Neuron Diseases, WFN ALS Clinical Trials Consortium, WFN Research Committees	Probable ALS: UMN and LMN signs in at least two regions, with some UMN signs rostral to LMN signs. Possible ALS: UMN and LMN signs in only one region, or UMN signs alone in two or more regions, or LMN signs rostral to UMN signs. Laboratory-supported probable ALS: Clinical evidence of UMN and LMN signs in only one region, or UMN signs alone in one region and neuroelectrophysiological evidence of LMN signs in at least two regions. Definite ALS: UMN and LMN signs in the bulbar region and at least two spinal regions, or UMN signs in at least two spinal regions and LMN signs in three spinal regions [2].	The possible, probable, probable laboratory supported and definite ALS depending on the number of affected segments combined with clinical and/or neuroelectrophysiological findings [5–7].	Diagnostic sensitivity was partially improved via removing the ‘suspected’ category and adding a ‘laboratory-supported probable” category. But the diagnostic sensitivity and delay, and limiting recruitment remained a challenge, particularly in the early stages. The specificity of the criteria was also the highest [5–7].
Awaji criteria or Awaji-shima criteria [3]	2008	Awaji, Japan	A consensus meeting of major neurologists from European, United States and Japan	Probable ALS: Clinical or neuroelectrophysiological evidence of UMN and LMN signs in at least two regions, with some UMN signs rostral to LMN signs. Possible ALS: Clinical or neuroelectrophysiological evidence of UMN and LMN signs in only one region, or UMN signs alone in two or more regions, or LMN signs rostral to UMN signs. Definite ALS: Clinical or neuroelectrophysiological evidence of UMN and LMN signs in the bulbar region and at least two spinal regions, or UMN and LMN signs in three spinal region [3].	The novel neuroelectrophysiological features of LMN dysfunction including the acute changes such as fibrillation potentials and the chronic neurogenic changes such as unstable motor units, were considered to be equivalent to the clinical features of LMN dysfunction. Separately, fasciculations were also identified as the features of active denervation morphology used to define the ALS-specific fasciculations. This criteria further emphasized and extended the useful of neuroelectrophysiological examination in diagnosing this disease on the base of Airlie House criteria [5–7].	Largely recommended neuroelectrophysiological data in diagnosing ALS. The diagnostic sensitivity and delay, and the limiting recruitment compared with 2000 revised criteria were higher. The increased diagnostic certainty and earlier diagnosis rate without increasing the false-positive rate of LMN damage. The specificity of the criteria was also higher, but increased a small amount of potential false-positive rate of this disease [5–7].
Gold Coast criteria [4]	September 27–29, 2019	Gold Coast, Australia	The International Federation of Clinical Neurophysiology, the World Federation of Neurology, the ALS Association, and the MND Association	Progressive motor impairment, documented by history or repeated clinical assessment, preceded by normal motor function. Upper and lower motor neuron dysfunction in at least one body region, or lower motor neuron dysfunction in at least two body regions. Investigative findings that exclude alternative diseases [4].	The simplified criteria abandoned the previous diagnostic categories of possible, probable and definite [4].	The new criteria facilitated to definitively diagnosis ALS in the early stages. Simplified the diagnostic program, increased the diagnostic sensitivity, and dispelled the uncertainty and confusion for patients and their families. But increased the potential false-positive rate of this disease also was challenge. The specificity of the criteria was the lowest in all current diagnostic criteria because of increasing the potential false-positive rate of this disease [4–7].

[1] Brooks BR. J Neurol Sci. 1994 Jul:124 Suppl: 96–107. doi: 10.1016/0022-510x(94)90191-0. [2] Brooks et al. Amyotroph Lateral Scler Other Motor Neuron Disord. 2000 Dec;1(5):293–9. doi: 10.1080/146608200300079536. [3] Carvalho et al. Clin Neurophysiol. 2008 Mar;119(3):497–503. doi: 10.1016/j.clinph.2007.09.143. [4] Shefner et al. Clin Neurophysiol. 2020 Aug;131(8):1975–1978. doi:10.1016/j.clinph.2020.04.005. [5] Feldman et al. Lancet. 2022 October 15;400(10360):1363–1380. doi:10.1016/S0140-6736(22)01272-7. [6] Goutman et al. Lancet Neurol. 2022 May;21(5):480–493. doi: 10.1016/S1474-4422(21)00465-8. [7] Al-Chalabi et al. Lancet Neurol. 2016 Oct;15(11):1182–94. doi: 10.1016/S1474-4422(16)30199-5. *Abbreviation*: UMN: upper motor neuron; LMN: lower motor neuron. Definitions of UMN and LMN damaged signs and phenotypes. UMN: Betz cells in layer 5 of the primary motor cortex. UMN dysfunction is characterized by the spasticity, hyperreflexia and the pathological reflexes (including Hoffmann, Babinski, snout). LMN: Brainstem cranial motor nerve nuclei or anterior horn cells. LMN dysfunction is characterized by muscle weakness, atrophy and fasciculations.

### 4.1. Doubt about the diagnostic criteria of ALS

The World Federation of Neurology Research Group on Motor Neuron Diseases formulated the first diagnostic criteria of ALS in 1994, El Escorial diagnostic criteria [40]. The El Escorial diagnostic criteria were revised in 2000, known as the Airlie House criteria, which modifies and improves the clinical diagnostic parameters, improving the ALS clinical diagnostic ratio, and differentiating the ALS diagnostic criteria for the clinical investigation from that for drug trials, which is benefit to the conduction of clinical trials [41]. The revised Airlie House criteria classified ALS into four different categories of clinically definite, clinically probable, clinically probable-laboratory supported and clinically possible according to the diagnostic criteria. It is noted that the patients with the pure LMN syndrome in the classification of El Escorial diagnostic criteria were classified into the clinically suspected category, however, this category was deleted from the revised Airlie House criteria. Thus, a significant number of patients who either have a pure LMN syndrome or who early in the course of the disease don’t have obvious UMN signs undoubtedly will be excluded in the revised criteria. Therefore, the Airlie House criteria probably increase more clinical diagnostic error ratio of ALS, isn’t very profit to clinical practice. The revised El Escorial Criteria in Awaji further simplified the diagnostic criteria on ALS [42]. Up to now, although the ALS diagnostic criteria have been revised for many times, the credible and accurate objective diagnostic parameters like the biological markers for helpful and useful clinical exact diagnosis haven’t been found yet [8–10] (Table 1).

The classical ALS diagnostic criteria including El Escorial [40,43,44], Airlie House [45] and Awaji diagnostic [46] criteria, which differentiates the classifications of ALS from possible to probable to definite ALS based on the number of affected segments combined with clinical and/or neuroelectrophysiological findings. The disadvantages are to delay early diagnosis and hinder the recruit patients in the clinical trial, because these nomenclature usually are misinterpreted the unlikely or inaccurate diagnosis to delay the early diagnosis and therapeutics of ALS. In fact, almost all patients diagnosed as possible and probable ALS patients might progress ultimately the definite ALS. In order to address these limitations, an emerging diagnostic criteria, the Gold Coast criteria [6], was reformulated to improve the diagnostic parameters for ALS, particularly in the early stages of disease when clinical symptoms are slight. The diagnostic criteria deleted the diagnostic categories of possible, probable, definite classification and excluded primary lateral sclerosis as a form of ALS and added the subtype of progressive muscular atrophy. The Gold Coast criteria facilitate diagnosing ALS early. However, it is importantly noted that the specificity of Gold Coast criteria is relatively less. For example, it might ignore the patients exhibiting primary lateral sclerosis at the onset early stage and ultimately developing ALS. In general, the current diagnostic criteria exist their advantages and disadvantage [47]. The optimal diagnostic criteria need further carefully consult each other among the neurologist yet.

The current diagnostic criteria of ALS [8,41] has conducted to definite mainly depending on the clinical manifestations yet, hasn’t any objective auxiliary diagnostic technology to definite the clinical signs of ALS besides electromyogram, especially the signs of cortical spinal lateral tract damage. The other clinical auxiliary examined methods including the computerized tomographic scanning, the magnetic resonance imaging (MRI) and some laboratory tests only are used in the differential diagnosis to exclude the ALS similar diseases. However, in the clinical practices of ALS diagnosis, the electromyogram only detects the damage of LMD, up to now, although some investigated results about examining the damage of UMD including the neuroimaging study like the functional MRI and positron emission tomography as well as transcranial ultrasound examination and transcranial magnetic stimulation (e.g. central motor conduction time, triple stimulation) in all diagnostic criteria for detecting UMN involvement in ALS patients were report to be helpful to identify and find the damage of UMD [8,48], while the objective and defined diagnostic evidences about the UMD damage haven’t been found yet. At present, the diagnosis of UMD still depends on the clinical manifestations, which mainly includes spasticity, hyperreflexia and the pathological signs like the Babinski sign. It is necessary for diagnosing ALS that both UMD and LMD are simultaneously damaged in the current diagnostic criteria. Based on the current diagnostic criteria, the diagnosis for some onset pattern of ALS such as the onset pattern by beginning with the muscle atrophy innervated by spinal cord exists problem, it means that if the LMD in four limbs were seriously damaged, the motor neurons in the anterior horn almost completely died, then began to damage UMD, the UMD damaged clinical manifestations of the increased muscle tension, the hyperfunction of tendon reflexes and the pathological signs can’t occur because of the broken of reflex arc, the potential damage of UMD can’t be clinically detected because that the signs of UMD damage are completely covered by the serious LMD lesion, for this onset type of ALS, the current diagnostic criteria for ALS obviously isn’t adaptive. Therefore, need modify and improve [2].

## 5. Commentary and perspective

The current nomenclature and diagnostic criteria of ALS still exists doubt, which brings some troubles and difficulties in the clinical study, diagnosis and early treatment of ALS. Therefore, I suggest that the nomenclature and diagnostic criteria of ALS should been comprehensively discussed and further revised. How to make the nomenclature of ALS and improve the early and accurate diagnosis of ALS is an important and urgent scientific task. In America, ALS usually is known as Lou Gerhrig’s disease because of a famous baseball player in American history, was diagnosed as ALS on June 21, 1939, ALS is named Lou Gerhrig’s disease in order to memory him [49]. Therefore, whether or not could use Lou Gerhrig name to replace ALS. Such as Alzheimer’s disease and Parkinson’s disease, these diseases use the names of the medical scientists who first described or found this disease to name the disease in order to memory these famous persons. Of course, at the international unwritten traditional convention, the disease nomenclature often is named based on the name of scientist who firstly described or found this disease, or who provided some important contributions for this disease. Regarding to use the nomenclature of the famous not scientist name hasn’t been reported up to date to the best of my knowledge in the history of international medical nomenclature. Therefore, the nomenclature of ALS need be drafted and proposed the nomenclature of ALS after comprehensively discussing among neurologists, and the more accurate nomenclature about ALS need extensively discuss by neurologist to search a most appropriate word.

In addition, how to improve the early and accurate diagnosis of ALS is a problem that should be solved quickly yet, which is the reason that no any effective treatment currently can use in the clinical prevent and cure ALS, is waiting for further exploring and investigating the effective prevention and/or cure of ALS based on the early and accurate diagnosis. Based on author’s research experiences, author suggest that it might be a promising and feasible method searching the effective diagnostic biomarkers through retrospectively surveying the ALS patients after the accurate diagnostic of ALS by performing extensive autopsy to improve the ALS diagnosis.

Moreover, the heterogeneity of ALS is very various and complex [50,51], among them, the genetic heterogeneity is very obvious, a series of genes mutations have been found participated in the pathogenesis of ALS [5]. Besides, the phenotypic, etiologic and mechanistic heterogeneities also are relatively complex and variable, which requires us to comprehensively consider the various aspects of ALS complex heterogeneities when proposing the most propriety namelature of ALS. Meanwhile, the diagnosis more should be like namelature to extensively consider the genetics, phenotypic, etiologic and mechanistic heterogeneities to formulate the accurate diagnostic criteria, couldn’t only simply depend on the clinical manifestations and onset location like the current diagnostic criteria [8].

## Data Availability

Data sharing is not applicable to this article as no new data were created or analyzed in this study.
